# Capillary Rarefaction in Obesity and Metabolic Diseases—Organ-Specificity and Possible Mechanisms

**DOI:** 10.3390/cells9122683

**Published:** 2020-12-14

**Authors:** Satu Paavonsalo, Sangeetha Hariharan, Madeleine H. Lackman, Sinem Karaman

**Affiliations:** 1 Wihuri Research Institute, 00290 Helsinki, Finland; satu.paavonsalo@helsinki.fi; 2 Translational Cancer Medicine Research Program, Faculty of Medicine, University of Helsinki, 00014 Helsinki, Finland; 3 Individualized Drug Therapy Research Program, Faculty of Medicine, University of Helsinki, 00014 Helsinki, Finland; sangeetha.hariharan@helsinki.fi (S.H.); madeleine.lackman@helsinki.fi (M.H.L.)

**Keywords:** capillary rarefaction, endothelial cell, organotypic vasculature, obesity, VEGFR signaling

## Abstract

Obesity and its comorbidities like diabetes, hypertension and other cardiovascular disorders are the leading causes of death and disability worldwide. Metabolic diseases cause vascular dysfunction and loss of capillaries termed capillary rarefaction. Interestingly, obesity seems to affect capillary beds in an organ-specific manner, causing morphological and functional changes in some tissues but not in others. Accordingly, treatment strategies targeting capillary rarefaction result in distinct outcomes depending on the organ. In recent years, organ-specific vasculature and endothelial heterogeneity have been in the spotlight in the field of vascular biology since specialized vascular systems have been shown to contribute to organ function by secreting varying autocrine and paracrine factors and by providing niches for stem cells. This review summarizes the recent literature covering studies on organ-specific capillary rarefaction observed in obesity and metabolic diseases and explores the underlying mechanisms, with multiple modes of action proposed. It also provides a glimpse of the reported therapeutic perspectives targeting capillary rarefaction. Further studies should address the reasons for such organ-specificity of capillary rarefaction, investigate strategies for its prevention and reversibility and examine potential signaling pathways that can be exploited to target it.

## 1. Introduction

The capillary network’s main function is to enable proper tissue metabolism by delivering oxygen and nutrients from the blood into tissues and removing carbon dioxide and waste products from the tissues into circulation. Capillaries can also have other, specialized functions in tissues that further recondition the blood by, for example, storing and recycling fat (adipose tissue (AT) capillaries) or by clearing waste products and mediating blood composition (renal capillaries). Obesity, one of the largest health problems globally, is defined as excessive fat accumulation that presents a risk to health with a body mass index (BMI) value over 30 kg/m^2^. Obesity and its associated metabolic diseases are linked to vascular dysfunction and loss of capillaries termed as “capillary rarefaction”. Due to the tissue-specific features and functions of different capillary networks, obesity-induced capillary rarefaction may contribute to different systemic consequences depending on the affected tissue. For instance, capillary rarefaction in skeletal muscle may impact glucose homeostasis, while rarefaction in pancreatic islets can cause insufficient insulin secretion to the blood circulation. In this review, we have a specific focus on obesity studies in human subjects and animal models but we also mention results from obesity-associated diseases and conditions, including diabetes and hypertension.

## 2. Obesity-Induced Capillary Rarefaction in Different Tissues

**Figure 1:**
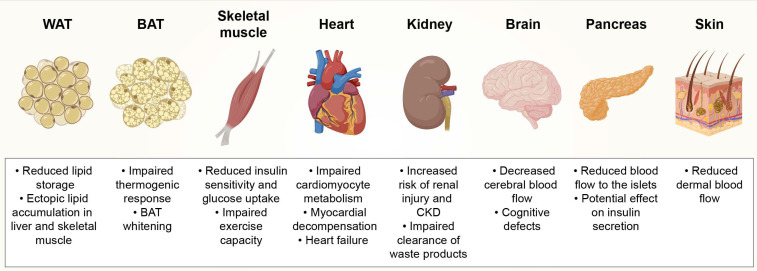
Consequences of obesity-induced capillary rarefaction on tissue function. White adipose tissue (WAT), brown adipose tissue (BAT), chronic kidney disease (CKD).

Obesity impairs the microcirculation in different organs, causing morphological (rarefaction and remodeling) and functional (dilation and recruitment) alterations in the capillary networks. It is important to take into account that changes in capillary densities (number of capillaries/tissue area or volume) might not necessarily reflect anatomical capillary rarefaction (number of capillaries/parenchymal cell). The difference between these two measurements is especially significant for tissues where obesity causes enlargement of the parenchymal cells, such as muscle fibers or adipocytes, which pushes capillaries away from each other. As such, the reader should consider the reported results in the light of how capillary rarefaction was measured in the respective organ, which we have indicated in brackets. Previously reported consequences of obesity-induced capillary rarefaction in various tissues are summarized in Figure 1.

### 2.1. Adipose Tissue (AT)

AT functions as a lipid storing depot that also secretes autocrine, paracrine and endocrine factors and participates in the systemic regulation of metabolism [1]. AT can be divided into distinct depots based on their location, structure and function. Below, we will summarize findings on obesity-induced capillary rarefaction in the two main AT types that are white and brown AT (WAT and BAT, respectively).

White adipose tissue (WAT) is the most common type of AT and it is distributed throughout the body. In addition to its role in lipid storage, WAT also provides insulation under the skin (subcutaneous WAT (sWAT)) and cushioning around organs (visceral WAT (vWAT)) [2]. The adipocytes in WAT have a single, large lipid droplet from which the stored triglycerides can be hydrolyzed into free fatty acids (FFAs) that are released into the bloodstream to supply other tissues during periods of high energy demand [2]. The energy surplus in obesity results in the expansion of WAT through increased adipocyte size (hypertrophy) and adipocyte proliferation (hyperplasia) [3]. However, the capillary networks in WAT fail to match their growth with the rapid expansion of WAT depots, but, instead are reported to undergo rarefaction upon obesity.

Several reports indicate an association between human obesity and WAT capillary rarefaction. Immunofluorescence (IF) analyses have shown decreased capillary density in the sWAT (number of capillaries/tissue area and number of capillaries/adipocyte) [4] and vWAT (number of capillaries/tissue area) [5] of patients with obesity compared to lean subjects. Similarly, immunohistochemical (IHC) analyses showed lower capillary density (number of capillaries/tissue area) in the sWAT and vWAT of normoglycemic, prediabetic and type 2 diabetes mellitus (T2DM) patients with obesity compared to lean subjects [6]. Moreover, IHC analyses showed lower capillary density (number of capillaries/tissue area and number of capillaries/adipocyte) in the sWAT of patients with obesity compared to overweight patients and ex vivo experiments revealed reduced number of capillary branches in sWAT explants from patients with obesity compared to explants from overweight patients [7]. These results suggest that sWAT’s angiogenic capacity decreases with morbid obesity [7]. In line with human studies, IF and IHC analyses showed that diet-induced obesity led to decreased capillary density (number of capillaries/tissue area and number of capillaries/adipocyte) in the WAT of mice that were on a high-fat, high-sucrose (HFHS) diet for eight weeks [8]. A reduction in capillary density (number of capillaries/tissue area and number of capillaries/adipocyte) was also observed in the vWAT of *ob*/*ob* mice, which lack functional leptin and are used as a genetic model of obesity, although the rarefaction phenotype was not as clear as in diet-induced obese mice [8].

The major pathologies associated with obesity-induced capillary rarefaction in WAT include inflammation, necrosis and systemic metabolic dysfunction [5,7,8,9,10]. Obesity-induced capillary rarefaction in WAT seems to be especially harmful when occurring in the sWAT depots that are essential for long-term storage of the excess energy in the form of triglycerides [7]. If the capillarization in sWAT is insufficient, sWAT’s capacity to store lipids becomes compromised and lipids start to accumulate in other tissues, such as liver, skeletal muscle and vWAT, which is associated with increased risk of developing insulin resistance and T2DM [11,12,13] (Figure 1).

In contrast to WAT, brown adipose tissue (BAT) has a much more limited distribution in the body. BAT is located mainly around adrenal glands, great vessels and the neck region [2]. The adipocytes in BAT contain multilocular lipid droplets and numerous large mitochondria, conferring BAT with a brown color. The primary role of BAT is to generate heat by non-shivering thermogenesis through activation via the sympathetic nervous system [14]. Importantly, BAT is abundantly present in hibernating animals and infants that require non-shivering thermogenesis and the density of active BAT decreases with aging and obesity (reviewed in Reference [15]). The reduction in active BAT density upon obesity is associated with enlarged lipid droplets in BAT adipocytes, a phenotypical shift towards “white adipocyte” [8] and capillary rarefaction.

Although capillary rarefaction has not yet been reported in the BAT of patients with obesity (reviewed in Reference [15]), likely due to the massively reduced amount of BAT in adult humans, capillary rarefaction has been reported in the BAT of obese mice. IF and IHC analyses indicate reduced BAT capillary density (number of capillaries/tissue area and number of capillaries/adipocyte) in mice that were on HFHS diet for eight weeks [8]. Interestingly, the decrease in BAT capillary density could already be detected after one week of HFHS diet [8], reflecting the speed and robustness of capillary rarefaction in BAT compared to WAT. Lower capillary density (number of capillaries/tissue area and number of capillaries/adipocyte) in BAT was also observed in the genetically obese *ob*/*ob* mouse model, indicating that this effect seen in BAT depends more on obesity per se than on the type of diet [8].

Notably, obesity-induced capillary rarefaction and accumulation of enlarged lipid droplets in BAT adipocytes leads to a phenomenon called “BAT whitening” where these specialized adipocytes undergo phenotypic changes from brown to white adipocytes [8]. Associated with mitochondrial dysfunction, the whitening of BAT leads to the loss of primary BAT function, impairing thermogenic response that was confirmed by an acute cold-tolerance test [8] (Figure 1). Capillary rarefaction in BAT is also characterized by increased inflammation and systemic metabolic dysfunction [8,9].

In summary, growing evidence suggests that capillary rarefaction seen in WAT and BAT has systemic consequences beyond AT. These include lipid spillover, reduced thermogenic response, inflammation and perturbed metabolism, pinpointing the importance of maintaining vascular density and function in these tissues.

### 2.2. Skeletal Muscle

The capillary network in skeletal muscle enables the transport of oxygen and nutrients to fulfill the muscle fiber’s metabolic requirements, especially during exercise. Notably, skeletal muscle plays a pivotal role in glucose clearance and the capillary network is responsible for approximately 70%–90% of the insulin-stimulated whole-body glucose uptake [16,17]. Obesity is associated with reduced blood flow, muscle fiber hypertrophy and decreased capillary density in skeletal muscle, suggesting that obesity disrupts the angiogenic response to muscle fiber hypertrophy, resulting in capillary rarefaction [18,19,20].

Using a modified adenosine triphosphatase (ATPase) method for simultaneously staining capillaries and providing fiber typing (type I and II), Gavin et al., found lower capillary density (number of capillaries/tissue area) in patients with obesity compared to lean subjects [19]. Although, it should be noted that studies with more selective capillary markers are required to confirm anatomical and functional capillary rarefaction in patients with obesity. Obesity-induced capillary rarefaction in skeletal muscle has also been observed in animal models, especially later on during the lifespan. A high-fat diet (HFD) regimen of six weeks did not lead to capillary rarefaction in the skeletal muscle of wild-type rats based on IF and side-stream darkfield imaging analyses, indicated by unchanged capillary volume and similar amount of functional capillaries (number of perfused capillaries/tissue area) [21]. However, reduced capillary density (number of capillaries/tissue area) was observed already in seven-week-old obese Zucker rats that are used as a model of genetic obesity and diabetes [22]. During a 20-week-long lifespan of this rat model, the onset of capillary rarefaction was seen to occur in two phases: the early phase between seven to ten weeks of age and the later phase between 13–17 weeks of age [22]. The early phase was associated with altered venular function and early elevation in oxidative stress, tumor necrosis factor (TNF)-α levels and vascular production of thromboxane A_2_, whereas the later phase was associated with loss of nitric oxide (NO) bioavailability [22]. These data collectively suggest that rarefaction in the skeletal muscle is initiated at the venular capillaries and continues to proceed towards arteriolar capillaries. Obesity-induced capillary rarefaction in the skeletal muscle is also associated with impaired exercise capacity [19] and may decrease resting blood flow [18,20] (Figure 1).

### 2.3. Heart

To ensure a sufficient supply of oxygen for the significant metabolic needs of the myocardium, cardiac capillaries are arranged densely so that every cardiomyocyte lies within a short distance of at least one capillary. The effects of obesity on the myocardium seem to vary, as some studies report obesity-associated cardiomyocyte hypertrophy [23,24] and others do not [25] and the cardiac capillary density in obesity has been reported to be either increased or decreased in different studies.

IHC analyses show lower capillary density (capillary length/tissue volume) in the left ventricle of patients with obesity compared to lean subjects [26]. Obesity-induced coronary capillary rarefaction has also been reported in animal models. Based on IHC analyses, obese Zucker rats had lower capillary density (number of capillaries/tissue area and number of capillaries/cardiomyocyte) in the myocardium than lean Zucker rats at eight months of age [23]. Similarly, IF analyses showed that obese Wistar-Kyoto rats with metabolic syndrome fed with HFD for 32 weeks had lower capillary density (number of capillaries/tissue area) in the left ventricle compared to rats on normal chow [27]. Six and 12 months old obese diabetic *db/db* mice that carry an inactivating mutation on the leptin receptor gene had lower myocardial capillary density (number of capillaries/tissue area) than the lean wild-type mice [28]. In contrast to these studies, IHC analyses have shown that Otsuka Long-Evans Tokushima fatty rats had increased sub-endocardial capillary density (number of capillaries/tissue area) after 20 weeks of age but this increase was lost at 40 and 60 weeks of age [29]. These results suggest that upon obesity, there may be an initial increase in cardiac capillary density, followed by capillary rarefaction as the obese state progresses.

In addition to obesity, diabetes mellitus (DM) can cause microcirculatory rarefaction and impair the responsiveness of ischemic myocardium to proangiogenic factors. Hinkel and colleagues compared the myocardium of hearts from end-stage patients with and without DM undergoing heart transplantation, utilizing specimens of freshly explanted hearts for histological and functional analyses. Their findings point to distinct capillary rarefaction by lower capillary density (number of capillaries/tissue area) and pericyte loss in hearts derived from patients with DM, accompanied by a lowered angiopoietin 1 (ANGPT1)/ANGPT2 ratio in the DM group [30].

Since oxygen extraction is constantly close to maximum in the myocardium [31], ischemia caused by capillary rarefaction can severely affect cardiac function. Obesity-induced rarefaction in cardiac capillaries may contribute to impaired myocardial blood flow, defects in cardiomyocyte metabolism, diastolic dysfunction and elevated risk of heart failure [32,33,34,35,36] (Figure 1). Although the results from these studies suggest that obesity and its related comorbidities such as diabetes may result in capillary rarefaction in the heart, further studies in humans and animal models are warranted to underpin the timing and the extent of obesity- and diabetes-induced capillary rarefaction in the myocardium.

### 2.4. Kidneys

The distinct capillary networks in the kidneys, including glomerular and cortical peritubular microcirculation, are pivotal in maintaining homeostasis by modulating blood composition, volume, pH and acid-base balance [37]. Glomerular capillaries filter the blood flowing through the kidneys, whereas peritubular capillaries reabsorb water, nutrients, hormones and other substances from the glomerular filtrate back into systemic circulation. The excess lipids in obesity accumulate in and around the kidneys, promoting the establishment of “fatty kidney” and a pro-inflammatory state with oxidative stress, endothelial cell (EC) dysfunction and capillary remodeling [38,39]. The effects of obesity on renal capillary densities seem to differ depending on the type of capillary, increasing the density of glomerular capillaries and causing rarefaction of the peritubular capillaries.

During the early stages, obesity may initially cause increased glomerular hydrostatic pressure and tubular reabsorption, leading to glomerular hypertrophy and enhanced capillarization in the kidney [40]. Furthermore, obesity is found to increase the capillary density (number of capillaries/tissue area) in the outer cortex of the kidney in diet-induced obese pigs with metabolic syndrome, shown by micro-computed tomography (micro-CT) analyses [41]. The initial increase in renal microvascularization in obesity may be due to inflammation promoted by the deposition of lipids in and around the kidneys [38,42]. However, obesity-induced neo-vascularization might be only temporary because the newly-formed vessels are likely to be unstable and the prolonged increase in glomerular hydrostatic pressure and EC dysfunction will eventually lead to renal capillary injury and rarefaction [43,44]. In contrast to the glomerular vasculature, lower capillary density (capillary area/tissue area) has been reported in the peritubular areas of the kidneys in obese mice after 12 weeks of HFD feeding, shown by IHC analyses [45,46] and electron microscopy [46]. In obese pigs, micro-CT showed decreased capillary density (number of capillaries/tissue area) in the kidneys of obese pigs with renal artery stenosis (RAS) when compared to lean pigs with RAS, suggesting that obesity aggravates capillary loss in the kidneys of large animal models [47].

The loss of glomerular capillary function and capillary rarefaction reduces peritubular blood flow and leads to ischemia and injury in the renal tubules and parenchyma in humans [48,49,50]. Even though capillary rarefaction may develop slowly in the kidneys, it is suggested to be an important mediator in the progression of renal injury and chronic kidney disease (CKD) in many patients with obesity [39,51] (Figure 1). In addition to participating in CKD progression, obesity-induced rarefaction in the kidneys may also contribute to microvascular dysfunction in other tissues as the renal capillary networks fail to clear waste products and adipokines from the body, increasing the amount of the so-called uremic toxins [52] (Figure 1).

### 2.5. Brain

Proper brain function requires that the cerebral blood flow is constant despite the metabolic demands of other organs and fluctuations in blood pressure [53]. Obesity is associated with decreased cerebral blood flow [54,55,56] and there are varying reports on obesity-induced capillary rarefaction depending on the distinct regions of the brain analyzed. Compared to lean Zucker rats, obese Zucker rats present progressive capillary rarefaction by decreased cerebral cortical capillary density (number of capillaries/tissue area) that is not observed at the age of seven to eight weeks but is detected at the age of 12–13 weeks and further progressing at the age of 16–17 weeks, as shown by IHC analyses [57]. Similarly, IF analyses showed no changes in capillary densities (capillary area/tissue area) in the hippocampus of obese rats that were on HFD for six months [58]. On the other hand, obese Wistar-Kyoto rats with metabolic syndrome had decreased capillary density in the cerebral cortex after 20 weeks of HFD feeding compared to the lean rats on chow diet, as shown by intravital video microscopy [59]. Additionally, IF analyses showed that compared to the lean mice, five months of HFD feeding resulted in decreased capillary density (capillary length/tissue volume) in both the cortex and hippocampus of aged, 24-month-old mice but not in younger, seven-month-old mice [60]. These results indicate that aging concurrent with obesity can aggravate capillary rarefaction in the brain, which likely contributes to cognitive decline in older patients with obesity [60] (Figure 1). In addition to cognitive defects, obesity has been suggested to increase the risk of stroke [61] and dementia [62]. However, it needs to be clarified whether obesity-induced capillary rarefaction plays a role in the development of these pathologies.

### 2.6. Pancreas

Pancreas is composed of two structurally and functionally distinct tissues, namely the endocrine and exocrine pancreas. The endocrine pancreas contains Langerhans islets that produce hormones, including insulin, glucagon and ghrelin, while the exocrine pancreas consists of ductal and acinar epithelial cells which make up the majority of the pancreatic mass [63]. Obesity has been reported to cause capillary rarefaction in the specialized parts of the pancreas in animal models. In a transgenic human islet amyloid polypeptide (HIP) rat model of T2DM, transmission electron microscopy (TEM) showed capillary rarefaction of intra-islet capillaries in 14-month-old rats compared to control rats [64]. In contrast to rarefaction of intra-islet capillaries, there was enhanced angiogenesis in the peri-islet capillaries [64]. However, these results were only based on visual observations and as such, quantifications of capillary densities are required to draw further conclusions on capillary rarefaction. In the same study, the authors suggested that pericyte apoptosis contributed to the intra-islet capillary rarefaction in this model, as pericytes seemed to be closely associated with collagenosis, intra-islet adipogenesis and peri-islet angiogenesis [64]. Similar to the rat model, *ob*/*ob* and HFD-induced mouse models of obesity had decreased intra-islet capillary density (number of capillaries/islet area) and enlarged intra-islet capillary diameter, whereas vessels in the exocrine tissue remained unaltered as shown by IF analyses [65]. Interestingly, in *ob*/*ob* mice, islet blood flow was lower than in lean mice when corrected for islet mass [66]. This suggested that despite having increased islet mass, the impaired blood flow in obese mice contributed to insufficient insulin secretion into bloodstream, causing hyperglycemia [66] (Figure 1). Altogether, these studies show that obesity leads to rarefaction of the intra-islet capillaries in the endocrine pancreas, while the exocrine pancreatic capillaries mostly remain unaltered.

### 2.7. Skin

The capillaries in the skin have an important and unique role in thermoregulation. In cold temperatures, dermal capillary blood flow is reduced by sympathetic impulses, whereas in warm temperatures, blood flow is enhanced to remove excess body heat [67]. Overweight and obesity have been associated with dermal capillary rarefaction and impaired capillary recruitment in the skin. Using reflectance-mode confocal microscopy, Altintas et al. found lower dermal capillary density (number of capillaries/tissue area) in overweight patients compared to lean subjects [68]. De Ciuceis et al. studied the additive effect of hypertension and weight loss in obesity-induced cutaneous capillary rarefaction using cutaneous intravital video-microscopy [69]. They reported that both normotensive and hypertensive patients with obesity have lower dermal capillary density (number of capillaries/tissue area) compared to controls [69]. However, weight loss following bariatric surgery in the same patients did not significantly improve capillary density, suggesting that obesity-induced capillary rarefaction in the skin might be long-lasting or even irreversible [69]. In contrast to the findings on obesity-induced dermal capillary rarefaction, Francischetti et al. did not observe changes in capillary densities (number of capillaries/tissue area) between lean subjects and patients with obesity (with and without metabolic syndrome) at resting state using cutaneous intravital video-microscopy [70]. However, evaluation of maximal skin capillary density with venous congestion and investigation of capillary recruitment during post-occlusive reactive hyperemia (PORH) showed that capillary density (number of capillaries/tissue area) was significantly higher in lean subjects than in patients with obesity [70]. This suggests that the capillaries are already maximally recruited in patients with obesity and metabolic syndrome at rest and thus, there is an absence of functional capillary reserve [70]. These data suggest that even though capillary rarefaction is observed in skin in obesity, its functional consequences still need to be evaluated in-depth.

## 3. Factors Regulating Capillary Density in Obesity

### 3.1. VEGF/VEGFR Signaling

**Figure 2:**
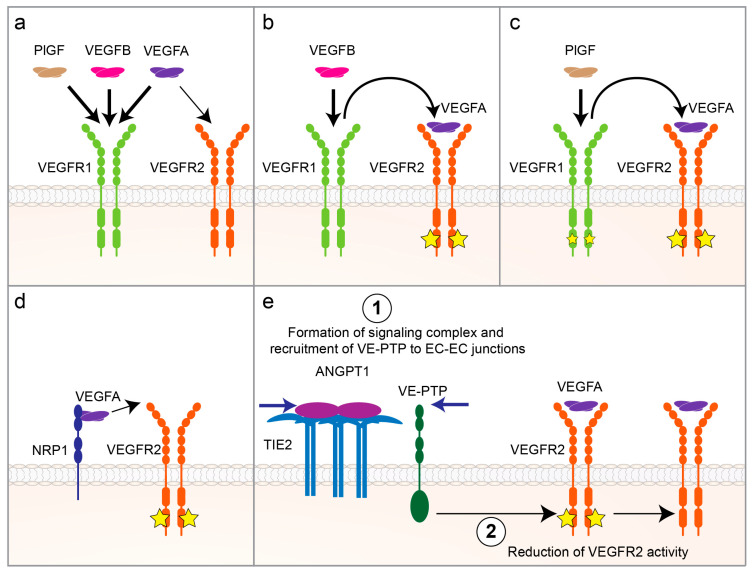
Regulation of vascular endothelial growth factor A (VEGFA)/ vascular endothelial growth factor receptor 2 (VEGFR2) signaling. (**a**) Placental growth factor (PlGF), VEGFB and VEGFA compete for binding to VEGFR1. VEGFA has ten times higher binding affinity to VEGFR1 than to VEGFR2 (reviewed in Reference [71]). (**b**) Binding of VEGFB to VEGFR1 displaces VEGFA and increases its bioavailability for binding to VEGFR2, which then induces angiogenic signaling via VEGFR2 activation. (**c**) Binding of PlGF to VEGFR1 increases angiogenesis both by displacing VEGFA and making it available for VEGFR2-binding and by activating PlGF’s own, albeit weaker, angiogenic signaling via VEGFR1 [77]. (**d**) Neuropilin-1 (NRP1) binds to VEGFA and presents it to VEGFR2, which enhances VEGFA/VEGFR2 signaling (reviewed in Reference [78]). (**e**) Binding of Angiopoietin 1 (ANGPT1) to its receptor TIE2 on endothelial cell (EC) surface activates signaling complex formation and recruits vascular endothelial protein tyrosine phosphatase (VE-PTP) to EC-EC junctions, which reduces VEGFR2 activity (reviewed in Reference [79]).

Vascular endothelial growth factors (VEGFs) and their cognate receptors (VEGFRs) are quintessential for the survival and maintenance of ECs [71]. Five VEGF ligands (VEGFA, -B, -C, -D and placental growth factor (PlGF)) bind to the VEGFRs on ECs to elicit various effects. Out of these ligands, VEGFA is the key growth factor for EC proliferation and survival via signaling through VEGFR2 (KDR) that is mainly expressed in blood vascular ECs. VEGFA also binds to VEGFR1 that is another receptor expressed predominantly by the blood vascular ECs and has a higher binding affinity for VEGFA than VEGFR2. However, VEGFR1′s weak tyrosine kinase activity suggests that it is a decoy receptor that negatively regulates VEGFA/VEGFR2 signaling (Figure 2a). On the other hand, VEGFB and PlGF compete with VEGFA for VEGFR1-binding, which then enhances VEGFA/VEGFR2 signaling by displacing VEGFA from VEGFR1 and making it available for VEGFR2 [72] (Figure 2b,c). Since VEGF/VEGFR signaling is pivotal for EC growth and survival, it has been the most extensively studied pathway in research on obesity-induced capillary rarefaction by using ligand/receptor deletions in genetic mouse models and ligand overexpression through transgenic mouse models or by the use of viral vectors.

**Figure 3:**
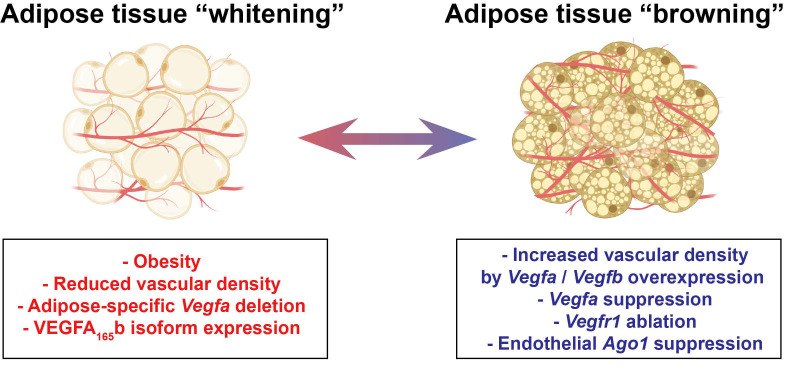
Endothelial regulation of adipose tissue (AT) phenotype. Factors such as diet-induced obesity [8], reduced vascular density due to a loss of *Vegfa* [8] or an increase in the anti-angiogenic isoform VEGFA_165_b may cause whitening of AT. On the other hand, increased vascular density due to *Vegfa* [74,75,83,84] or *Vegfb* [81] overexpression or *Vegfr1* ablation [82] can induce browning of WAT in mice. Interestingly, the suppression of *Vegfa* expression in mice also led to a browning phenotype in WAT, suggesting that further studies are required to conclude the exact role of VEGFA in this phenomenon. Recently, endothelial protein argonaute-1 (AGO1) suppression was also shown to increase vascular density and AT browning [85].

VEGFA is expressed at higher levels in BAT as compared to WAT and is essential for maintaining a higher capillary density required for BAT function [73]. Shimizu and colleagues studied VEGFA expression and its effect in WAT and BAT capillary densities in lean and obese mice [8]. In wild-type mice, eight-week-long HFHS diet caused notable capillary rarefaction in the WAT and BAT [8]. An AT-specific deletion of *Vegfa* in lean mice also resulted in capillary rarefaction in both WAT and BAT and the level of the reduction in capillary densities was similar to what was observed in mice with diet-induced obesity [8]. The decrease in BAT vasculature was strongly associated with reduced VEGFA levels in the tissue. Interestingly, the loss of capillaries due to *Vegfa* deletion also induced a “whitening” phenotype in BAT without diet-induced obesity, implicating the role of BAT vasculature in maintaining the brown adipocyte phenotype [8] (Figure 3). It remains to be addressed if this effect would be observed to the same extent in adult mice on HFHS diet, as the age of mice in the beginning of the experiment (four weeks) might also have contributed to the severity of the phenotypic outcome.

In contrast with the genetic deletion of *Vegfa*, both the constitutive overexpression and inducible overexpression of VEGFA resulted in increased vascular density in AT and protected the transgenic mice against HFD-induced obesity and insulin resistance [74,75]. It should also be noted that the effect of AT-specific overexpression of VEGFA on diet-induced obesity is partly due to the browning of WAT and the elevated thermogenesis in these models [74,75]. Suprisingly and in contrast with the other findings, the repression of *Vegfa* in adult mice also resulted in impressive browning of the epididymal WAT (eWAT) and a resistance to HFD-induced obesity, suggesting a role for VEGFA in energy metabolism, which is currently not completely understood [76] (Figure 3).

As opposed to its angiogenic isoforms, VEGFA also has inhibitory isoforms such as VEGFA_165_b, which are upregulated in obesity, counteracting the pro-angiogenic effects and promoting vascular rarefaction in the vWAT of patients with obesity [80]. VEGFA_165_b was shown to suppress the phosphorylation of the Y951 residue in VEGFR2, thereby impairing VEGFR2 signaling. The targeted blockade of VEGFA_165_b improved angiogenesis in vWAT, which partially explains the paradoxical decrease in capillary density concurrent with increased VEGFA levels in obesity. Hence, it is plausible that the obese milieu determines whether VEGFA acts as a compensatory or a pathological factor in the vasculature. This creates a challenge in determining if VEGFA is an optimal therapeutic target in obesity [80]. Thus, studies focusing on VEGFA levels should also consider the varying effects of distinct VEGFA isoforms, including the anti-angiogenic impact of VEGFA_165_b.

Another VEGF family member, VEGFB, has been shown to have potential to increase capillary density in various set-ups, without inducing the deleterious side effects of VEGFA. In one of such set-ups, adeno-associated virus (AAV)-mediated *Vegfb* transduction increased the capillary density in AT, induced AT browning and enhanced insulin delivery in the eWAT, heart and liver of HFD-fed mice [81]. In line with this, in a diet-induced obesity model, endothelial-specific *Vegfr1* deletion demonstrated a potent anti-obesity effect by improving global metabolism [82]. These findings further support the idea that high levels of VEGFB are tolerated better than high levels of VEGFA, since VEGFB acts by increasing the bioavailibility of physiological levels of VEGFA.

To summarize the findings from AT, the suppression of angiogenesis in AT results in different outcomes based on the timing of the intervention. Inhibiting angiogenesis before a diet-induced obesity protocol results in increased inflammation and hypoxia with a worse metabolic outcome ([9,75]), whereas blocking angiogenesis after obesity is already established has shown more beneficial effects [75]. On the contrary, increasing the capillarization of AT in either of these invervention scenarios seems to result in more favorable outcomes, indicating the importance of capillary coverage in AT ([9,81,82] and reviewed in Reference [86]).

In contrast to AT, VEGFA levels do not seem to contribute to capillary rarefaction in some other tissues during the progression of obesity. Lower capillary density but unaltered *VEGFA* levels in the skeletal muscle of patients with obesity have been reported in a human clinical trial, suggesting the role of other signaling pathways in obesity-induced capillary rarefaction [19]. Nevertheless, muscle-specific deletion of *Vegfa* has been shown to induce capillary rarefaction and to cause insulin resistance in skeletal muscle due to impaired perfusion [87], whereas the AAV-mediated delivery of *Vegfa* into skeletal muscle stimulated skeletal muscle regeneration in vivo in the short term [88]. However, one-year-long expression of VEGFA sustained by AAV-injection promoted aberrant angiogenesis and fibrosis in skeletal muscle [89], making therapies based on VEGFA usage less ideal and requiring strict dosing and timing of the VEGFA treatment. Furthermore, it remains to be studied if the VEGFA-induced neo-vasculature remains intact in the treated organ after VEGFA cessation.

It is worth mentioning that the VEGFA/VEGFR2 signaling pathway can also be modulated by co-receptors such as neuropilin-1 and 2 (NRP1 and NRP2, respectively) or via crosstalk with other signaling pathways such as ANGPT1/ANGPT1 receptor (TIE2). NRP1 and NRP2 are chief VEGFR co-receptors that bind VEGFs and present them to VEGFRs on the cell surface, thereby enhancing VEGFR signaling (Figure 2d). On the other hand, upon binding to its receptor TIE2, ANGPT1 can recruit vascular endothelial protein tyrosine phosphatase (VE-PTP) to EC-EC junctions and downregulate VEGFR2 signaling (Figure 2e). Therefore, the spatiotemporal differences in the expression patterns of these molecules could result in varying VEGF bioactivity in different tissues. We refer the reader to more in-depth reviews on these topics [78,79].

### 3.2. Inflammatory Mediators

Obesity causes notable changes in the levels of inflammatory mediators, which can contribute to capillary rarefaction. In this section, we summarize findings from studies that cover such inflammatory factors that have been shown to mediate capillary rarefaction in obesity.

#### 3.2.1. Soluble Factors

In a recent study, Koller and colleagues showed that proinflammatory mediators interleukin 1 β (IL1-β), TNF-α and thrombin can directly induce capillary tube regression of human umbilical vein ECs (HUVECs) in a 3D collagen regression assay [90]. In addition, prolonged inhibition of TNF-α reduced the severity of rarefaction, alleviated oxidative stress and decreased thromboxane A_2_ production, whereas inhibition of thromboxane A_2_ only blunted rarefaction. These results suggest that therapeutic prevention of inflammation and inhibition of thromboxane A_2_ production may impede capillary rarefaction [22]. On the other hand, Asterholm et al. describe that inflammation in AT is essential for its healthy expansion and even if the inflammatory mediators, such as TNF-α inhibit adipogenic differentiation in vitro, they are required for extracellular matrix (ECM) remodeling and angiogenesis in vivo [91]. These studies clearly show that the inflammatory pathways regulating capillary density may involve the same mediators but the effect of these mediators might be time- and context-dependent.

#### 3.2.2. Adhesion Molecules

Oxidative stress and inflammatory cytokines produced by perivascular fat upregulate the expression of adhesion molecules on the endothelium, enabling inflammatory cells to enter the tissue via the microcirculation [92,93]. In obese Zucker rats, the early phase of capillary rarefaction in skeletal muscle was associated with increased leukocyte adhesion and rolling [22]. One molecular mechanism underlying this phenomenon is the recruitment of adhesion molecules from the selectin and immunoglobulin families, that is, P-selectin, E-selectin, vascular cell adhesion molecule-1 (VCAM-1) and intercellular cell adhesion molecule-1 (ICAM-1). The upregulation of these molecules is linked to the upregulation of TNF-α [94,95]. Excessive levels of FFAs also upregulate P-selectin in post-capillary venules by reducing adenosine monophosphate-activated protein kinase (AMPK)/endothelial nitric oxide (eNO) signaling. Furthermore, the rolling leukocytes release myeloperoxidase (MPO), resulting in impaired adiponectin function [96].

#### 3.2.3. WNT5A

While Wnt family member 5A (WNT5A) induced the expression of proinflammatory cytokines from the macrophages and resulted in impaired insulin response in adipocytes, the genetic deletion of *Wnt5a* in mice did not result in reduced weight-gain or smaller adipocyte size but increased insulin sensitivity [97]. Interestingly, WNT5A can contribute to the loss of vasculature by increasing the expression of anti-angiogenic VEGFA_165b_ isoform [98,99].

#### 3.2.4. PlGF

PlGF binds to VEGFR1 and can induce angiogenesis and vasculogenesis by both displacing VEGFA and inducing its own, although weaker, angiogenic signaling via VEGFR1 [77] (Figure 2c). *Plgf* downregulation was previously linked to reduced blood vessel density in gonadal WAT (gWAT) and sWAT in obese PlGF-deficient mice fed with a HFD for 15 weeks [100]. PlGF was recently associated with enhanced inflammation and metabolic disorders in mice with HFD-induced obesity [101]. In this study, transgenic overexpression of *Plgf* increased the amount of type 1 and type 17 T helper cells (Th1 and Th17, respectively) in mice fed with a HFD for 16 weeks. Additionally, macrophage infiltration to eWAT and the levels of the proinflammatory cytokines IL-6, IL-17 and TNF-α were significantly increased in obese mice than in mice fed with a standard diet [101]. These results indicate that the inflammatory effects of PlGF overexpression counteract the beneficial outcomes of increased capillarization in HFD-induced obesity.

#### 3.2.5. Nitric Oxide (NO)

The bioavailability of nitric oxide (NO) was strongly linked to capillary rarefaction in obese Zucker rats upon metabolic syndrome. During chronic anti-cholesterol therapy, a particularly significant change was seen in the levels of the inflammatory markers chemokine C-C motif ligand 5 (CCL5), IL-10, monocyte chemoattractant protein-1 (MCP-1) and TNF-α, all of which correlated with decreased capillary density and a reduced NO bioavailability. Moreover, a chronic treatment with statins resulted in reduced inflammation and impeded rarefaction in the obese Zucker rat model [102].

#### 3.2.6. Apelin

Apelin is an endogenous peptide with several isoforms and it acts as a ligand for the apelin receptor (APJ) [103]. The apelin/APJ system was shown to promote a proangiogenic response in both human and murine ECs. The inflammatory markers TNF-α and hypoxia-inducible factor 1-alpha (HIF-1α) regulate apelin secretion and it is speculated that apelin has a role in anti-inflammatory pathways in obesity [104,105]. Many cell types, including adipocytes, secrete apelin and its expression is controversially upregulated in obesity. Apelin knockout (KO) mice are described to have decreased insulin sensitivity in skeletal muscle [106]. Based on its potential to increase vascular density, Yamamoto and colleagues tested the beneficial effects of apelin using a transgenic mouse model that overexpresses human apelin [107]. Indeed, overexpression of apelin in mice showed an increase in vascular mass in skeletal muscle and elevated oxygen consumption after a 20-week-long HFD feeding [107]. Additionally, the selective cyclooxygenase-2 (COX2) inhibitor celecoxib blocked angiogenesis and lymphangiogenesis in apelin-KO mice fed with a HFD for 17 weeks and promoted vessel stabilization, indicating that apelin is required for proper development of both blood and lymphatic vessels in AT [108].

### 3.3. MicroRNA (miRNA) Pathways

Post-transcriptional regulation of gene expression with various microRNAs (miRNAs) has also been reported in obesity-induced capillary rarefaction. In skeletal muscle, obesity downregulated the expression of miRNA-126 and activated phosphoinositide-3-kinase regulatory subunit 2 (*Pi3rR2*) expression. In obese Zucker rats, exercise training (ET) rescued the miRNA-126 expression levels and reversed the obesity-mediated capillary rarefaction, suggesting that the increased muscle contraction and activation of angiogenic signaling pathways by ET may play a role in maintaining normal capillary density [109]. Increased miRNA-16 expression was associated with the progression of capillary rarefaction in the hearts of the obese Zucker rats. Conversely, swimming exercise restored the levels of miRNA-16 and prevented capillary rarefaction [110]. The study in hypertension rat models by Fernandes et al. indicates the relevance of miRNA-16, -21 and -126 induced by ET in reducing blood pressure and preventing capillary rarefaction [111]. This study’s major new finding showcases how miRNA expression can be modulated by ET to vascularize the tissue and to treat capillary rarefaction and impaired angiogenesis during obesity-associated comorbidities, like hypertension.

In addition to individual miRNAs, *Argonaute 1* (AGO1), one of the miRNA-induced silencing complex components, has been shown to regulate AT vascular density and browning [85]. EC-specific *Ago1*-KO mice gained less fat mass, exhibited improved insulin sensitivity and energy metabolism concurrent with higher vascular density in sWAT and BAT when fed with a HFHS diet. These mice also had enhanced browning of sWAT and improved thermogenesis in BAT [85].

### 3.4. Other Factors

In this section, we discuss the potential factors that also seem to play a role in obesity-induced capillary rarefaction but do not fit into the categories discussed above.

#### 3.4.1. FOXO1

Forkhead box O transcription factor 1 (FOXO1) was recently shown to link the proliferative and metabolic pathways in ECs and its EC-specific deletion led to uncoordinated growth of vessels via elevated glycolysis and mitochondrial respiration, thereby contributing to the plasticity of ECs to respond to external stimuli [112]. In line with this, an EC-specific deletion of *Foxo1* in mice improved the proliferative capacity of ECs and prevented HFD-induced capillary rarefaction in AT [113].

#### 3.4.2. MAP4K4

Mitogen-activated protein kinase kinase kinase kinase 4 (MAP4K4) is a serine/threonine kinase that activates the mitogen-activated protein kinase 8 (MAPK8)/c-Jun N-terminal kinase (JNK) pathway and it is suggested to also play a role in TNF-α signaling. EC-specific *Map4k4* knockout (*M4k4^iECKO^*) mice displayed a trend towards improved glucose tolerance and significantly enhanced insulin sensitivity compared to controls after a 16-week-long HFD feeding [114]. When the capillary densities were assessed, the authors found that eWAT capillary densities were comparable between *M4k4^iECKO^* mice and the control littermates under both normal chow and HFD. Interestingly, the skeletal muscle of *M4k4^iECKO^* mice did not undergo capillary rarefaction during 16 weeks of HFD and the ECs isolated from these mice displayed enhanced energy metabolism and resistance to senescence, which could explain why these mice were more sensitive to insulin after HFD [114]. Despite this metabolic improvement, *M4k4^iECKO^* mice also displayed lymphatic vascular defects, as noted by chyle leakage in the abdomen (chylous ascites) and increased immune cell content in eWAT. Altogether, these results demonstrate a complex and critical role for endothelial MAP4K4 in maintaining lymphatic vascular integrity and promoting systemic insulin resistance in obesity [114].

#### 3.4.3. SIRT3

Sirtuin 3 (SIRT3) is a nicotinamide adenine dinucleotide (NAD)-dependent deacetylase associated with the mitochondria. Zeng and colleagues found that a 16-week-long HFD feeding significantly reduced the capillary density in the ventricles as compared to controls [115]. *Sirt3*-KO mice on normal diet also showed a strong reduction in cardiac capillary density. Even though HFD-fed mice had lower SIRT3 expression in the heart, HFD did not further worsen the capillary rarefaction observed in *Sirt3*-KO hearts, suggesting SIRT3 loss to be one of the pathways implicated in obesity-induced capillary rarefaction and SIRT3′s potential as a therapeutic target in preserving heart capillary density upon obesity [115].

#### 3.4.4. Thymosin-β4 (Tβ4)

Hinkel et al. have found that patients with DM undergoing heart transplantation displayed capillary rarefaction with a loss of pericytes in the myocardium [30]. *Ins^C94Y^* transgenic pigs, a model of permanent neonatal DM, were used to study how microvascular destabilization and neovascularization affect ischemic cardiomyopathy. Since thymosin-β4 (Tβ4) is a growth factor that induces both angiogenesis and pericyte coverage, they transduced the myocardium using a recombinant AAV (rAAV) encoding *Tβ4* (rAAV.Tβ4) 28 days after stent placement. rAAV.Tβ4 transduction significantly induced capillary growth and maturation in these diabetic pigs but to a lesser extent than in wild-type pigs. Furthermore, rAAV.Tβ4 transduction increased left-ventricular ejection fraction in the diabetic pigs but again to a lesser extent than in wild-type pigs. These results indicate that Tβ4 is a promising growth factor for the induction of neovascularization in ischemic hearts, even under diabetic conditions [30,116].

#### 3.4.5. Extracellular Matrix (ECM)

ECM remodeling and reconstitution are prerequisites for the hypertrophy and hyperplasia observed in obesity (reviewed in Reference [117]). ECM is one of the most important modulators of angiogenesis, not only because it determines a 3D matrix where the angiogenic sprouts are allowed to form but also because it retains several angiogenic factors and helps to guide sprouting [118]. Obesity can significantly alter the expression levels of various ECM components and modulate the angiogenic potential of AT (reviewed in Reference [119]). For instance, an increase in certain collagen variants can cause a reduction of capillary density in AT by increasing tissue stiffness, which does not favor adipocyte enlargement and EC proliferation [119]. These results suggest that ECM may regulate angiogenesis in AT.

## 4. Discussion

The results reported in studies investigating obesity-induced capillary rarefaction suggest that obesity causes diverse and tissue-specific effects on capillary networks depending on the organ studied. Importantly, obesity may not cause systemic capillary rarefaction since there are also tissues in which obesity-induced capillary rarefaction has not been reported so far. For instance, the capillaries in liver [120,121] and retina [122] do not seem to undergo obesity-induced capillary rarefaction. On the contrary, capillary density is affected by obesity in the tissues described in this review but the observed obesity-induced capillary rarefaction has tissue-specific features in its progression, depending on the duration and severity of the obese state. In general, obesity-induced capillary rarefaction seems to be initiated only after the obese state has been established for an extended period. There may even be a preceding phase in some tissues where capillary density is increased before capillary rarefaction is initiated, perhaps, suggesting a role for endothelial senescence. Additionally, capillary rarefaction progression can vary between phases of elevated rarefaction and phases of more subtle changes in the capillary networks.

There are numerous studies exploring the best ways to study obesity-induced capillary rarefaction. However, different groups report capillary rarefaction using different experimental set-ups and methods of measuring capillary density, which makes it challenging to interpret the results in a broader sense. Therefore, several parameters should be kept in mind when drawing conclusions about the effect of obesity on capillary networks. Firstly, majority of the current data regarding obesity-induced capillary rarefaction is obtained from animal studies. With animals, HFD-feeding can be used to induce weight gain to resemble human obesity but diet-induced phenotypes often vary among different studies depending on the chosen diet, the length of the study and the animals’ age. Thus, the results gained from animal studies should be validated in humans. Secondly, capillary density can be determined using different methods, out of which IHC and IF analyses seem to be the most common and reliable methods. In addition to these, capillary rarefaction has also been detected using intra-vital imaging in certain studies (e.g., References [64,123]) even though this method might not be precise enough to detect the smallest capillaries. Lastly, capillary density measurements may give different results depending on how the values are calculated. Capillary density values are commonly presented as the number of capillaries per tissue area. However, these quantifications do not take into account the effect which obesity may have on the areas surrounding the capillaries or on the size of the parenchymal cells. For example, the size of adipocytes and muscle fibers can increase upon obesity, thereby automatically decreasing the capillary density in a particular region of interest. Thus, a perhaps more reliable value for capillary coverage can be calculated as the number of capillaries per parenchymal cell. Moreover, Spencer et al. report that a reduction in the CD31+ capillary percentage was compensated by a 70% increase in larger vessels, resulting in a lack of change in total CD31+ vessel percentage in AT [4]. These findings additionally illustrate the fact that different types of vessels are differentially affected by obesity. It was also recently shown that an enlarged vascular area might not necessarily mean that there is angiogenesis in the sense of EC proliferation and in fact, the number of ECs might even be reduced after mitogenic stimuli [124]. Importantly, the functional consequences of capillary rarefaction might not only depend on the reduction of capillaries but also on their distribution in the tissues. Therefore, future work examining the effects of obesity on capillary networks of different organs of the same study subject is required to elucidate the initiation, progression, distribution and extent of capillary rarefaction in distinct organs.

The pathways that contribute to rarefaction mostly revolve around VEGF signaling, as VEGFA/VEGFR2 is one of the central pathways for EC survival. However, there are various studies showing that inflammatory factors, certain miRNAs and even changes in ECM composition also take part in obesity-induced rarefaction. In addition to these factors, obesity may cause capillary rarefaction via inducing other systemic conditions, such as hypertension [125]. Decreased capillary length density in the skeletal muscle of glucocorticoid-induced hypertensive Wistar rats has been connected to increased numbers of dead ECs in the mesentery and apoptotic ECs in the mesentery and skeletal muscle of the hypertensive rats, suggesting that hypertensive capillary rarefaction could result from enhanced EC apoptosis [126]. These findings suggest a link between hypertension and capillary rarefaction, possibly due to EC dysfunction and apoptosis. It is likely that hypertension also plays a significant part in obesity-induced capillary rarefaction, although it is difficult to determine the cause-effect relationship between hypertension and capillary rarefaction. Besides hypertension, the role of other factors including supporting cell types should also be considered as contributors in obesity-induced capillary rarefaction. Pericytes are mural cells that envelop vessels, maintain blood flow and provide stability to the vessel wall. In murine pancreatic islets, pericytes were shown to reduce capillary diameter and blood flow by sympathetic adrenergic input [127]. In pathological conditions, pericyte coverage can be lost (also called pericyte detachment), leading to vessel instability, altered capillary function and capillary rarefaction (reviewed in Reference [128]). HFD-induced obesity led to pericyte detachment in the AT of mice via increased PDGF-β expression of inflammatory macrophages [129]. In addition, pericyte coverage in pancreatic islets was lost in obese mice and in T2DM patients, suggesting that pericyte detachment might lead to inadequate insulin supply under these conditions [127]. These studies emphasize the importance of pericytes in regulating capillary density and function in obesity and metabolic diseases.

Regarding possible therapeutic approaches, there is currently no silver bullet in targeting obesity-induced capillary rarefaction. However, it is clear that this process is governed by different pathways, which creates opportunities to target capillary rarefaction separately in different tissues. For instance, ET has shown potential in preventing capillary rarefaction in skeletal muscle, heart and AT. In rats, ET prevents microvascular rarefaction in hypertension by balancing angiogenic and apoptotic factors [111]. In obese Wistar-Kyoto rats with metabolic syndrome, a moderate to high amount of ET was enough to reverse capillary rarefaction in the heart and skeletal muscle [27]. Dunford and colleagues have recently shown that diabetes-induced capillary rarefaction in skeletal muscle can be reversed with ET in rats. Moreover, ET-induced angiogenesis could be further enhanced with prazosin treatment resulting in additive angiogenesis, suggesting that multiple pathways could be targeted to achieve a superior angiogenic response [130]. In addition, prolonged ET has been shown to blunt microvascular rarefaction in the skeletal muscle of obese Zucker rats, potentially by increasing NO bioavailability [131]. In a recent human study, two-week-long sprint interval training was shown to increase the capillary density (capillary area/tissue area) and glucose uptake in abdominal sWAT in patients with insulin resistance but not in healthy patients [132]. Therefore, ET seems to be a viable strategy to alleviate capillary rarefaction in multiple tissues and further studies are needed to show the benefit of ET in other tissues that undergo rarefaction upon obesity and diabetes. In addition to ET, exenatide, which is a small molecule with anti-inflammatory properties, has also been reported to improve capillary rarefaction in obesity. After a four-week-long treatment, the exenatide-treated obese mice had reduced macrophage infiltration and adipocyte size, in addition to higher capillary density in AT [133]. These results suggest that inflammatory burden contributes to capillary rarefaction in AT and is at least partially reversible upon treatment with anti-inflammatory molecules. Although, it remains to be seen whether this is a direct effect on ECs or if it is an indirect effect due to the improved insulin sensitivity and the reduced size of the adipocytes.

In conclusion, the studies presented here suggest that obesity causes organ-specific capillary rarefaction and that the extent of capillary loss depends on the length and severity of the obese state. Our major exhortation for the readers is to keep in mind what the reported capillary rarefaction values refer to in each study. Based on what we summarize here, there seems to be a lack of studies that look at a variety of tissues simultaneously under the same experimental conditions in obesity and other metabolic disease models. There is also still ground to break on revealing how such organ-specificity is governed and whether the organ-specificity is due to the inherent characteristics of the ECs or due to the microenvironment provided by the tissue parenchyma. These questions could be answered with EC transplantation experiments. Since there seems to be several pathways involved in mediating capillary rarefaction, it may be possible to target these pathways separately in order to prevent or reverse capillary rarefaction more effectively. It should also be noted that, even though animal models share similarities with human disease, it is not completely clear if the capillary rarefaction observed in animal tissues resembles the pathologies observed in humans. Therefore, future studies are needed to help us better understand the mechanisms behind the organ-specificity of capillary rarefaction observed in obesity and to find links with other metabolic diseases sharing similar phenotypic manifestations.

## Abbreviations

AAV	adeno-associated virus
AGO1	argonaute 1
AMPK	adenosine monophosphate-activated protein kinase
ANGPT	angiopoietin
APJ	apelin receptor
AT	adipose tissue
ATPase	adenosine triphosphatase
BAT	brown adipose tissue
BMI	body mass index
CCL5	chemokine C-C motif ligand 5
CKD	chronic kidney disease
COX2	cyclooxygenase-2
DM	diabetes mellitus
EC	endothelial cell
ECM	extracellular matrix
eNO	endothelial nitric oxide
ET	exercise training
eWAT	epididymal white adipose tissue
FFA	free fatty acid
FOXO1	forkhead box O transcription factor 1
gWAT	gonadal white adipose tissue
HFD	high-fat diet
HFHS	high-fat, high-sucrose
HIF-1α	hypoxia-inducible factor 1-alpha
HUVEC	human umbilical vein endothelial cell
ICAM-1	intercellular cell adhesion molecule-1
IF	immunofluorescence
IHC	immunohistochemical
IL	interleukin
JNK	c-Jun N-terminal kinase
KO	knockout
MAP4K4	mitogen-activated protein kinase kinase kinase kinase 4
MAPK8	mitogen-activated protein kinase 8
MCP-1	monocyte chemoattractant protein-1
Micro-CT	micro-computed tomography
miRNA	microRNA
MPO	myeloperoxidase
NAD	nicotinamide adenine dinucleotide
NO	nitric oxide
NRP	neuropilin
Pi3rR2	phosphoinositide-3-kinase regulatory subunit 2
PlGF	placental growth factor
PORH	post-occlusive reactive hyperemia
rAAV	recombinant adeno-associated virus
rAAV.Tβ4	recombinant adeno-associated virus encoding thymosin-β4
RAS	renal artery stenosis
SIRT3	sirtuin 3
sWAT	subcutaneous white adipose tissue
T2DM	type 2 diabetes mellitus
TEM	transmission electron microscopy
Th1	type 1 T helper cell
Th17	type 17 T helper cell
TNF-α	tumor necrosis factor alpha
Tβ4	thymosin-beta 4
VCAM-1	vascular cell adhesion molecule-1
VE-PTP	vascular endothelial protein tyrosine phosphatase
VEGF	vascular endothelial growth factor
VEGFR	vascular endothelial growth factor receptor
vWAT	visceral white adipose tissue
WAT	white adipose tissue
WNT5A	Wingless-related integration site family member 5A
